# Low Back Pain in Athletes Is Associated with General and Sport Specific Risk Factors: A Comprehensive Review of Longitudinal Studies

**DOI:** 10.1155/2015/850184

**Published:** 2015-12-13

**Authors:** Vahideh Moradi, Amir-Hossein Memari, Monir ShayestehFar, Ramin Kordi

**Affiliations:** ^1^Department of Orthotics and Prosthetics, Faculty of Rehabilitation Sciences, Iran University of Medical Sciences, Tehran, Iran; ^2^Sports Medicine Research Center, Tehran University of Medical Sciences, Tehran, Iran

## Abstract

We aimed to examine systematically the available evidence on risk factors of low back pain (LBP) in athletes. We performed search without language restriction in PubMed, Ovid, Google Scholar, Scopus, and CINAHL. Longitudinal studies that examined possible risk factors of LBP in athletes were included in this systematic review. Based on methodological quality of studies, a best-evidence synthesis was conducted. Seven longitudinal studies were included, four of which had high methodological quality. Results showed that previous LBP, decreased lumbar flexion, and decreased lumbar extension are positively associated with LBP. There was moderate evidence for hip flexor tightness and high body weight as a risk factor. We found insufficient evidence for association between forward bending, previous injury, and amount of training per week, active years, age, and sex with LBP. In conclusion this study would provide a list of risk factors for LBP in athletes, though it showed a strong evidence for only a few including decrease lumbar flexion or extension, previous LBP, and high body weight. This review indicated a high heterogeneity of study characteristics including assessed risk factors and statistical techniques might limit the quality of evidence.

## 1. Introduction

Low back pain (LBP) is the most prevalent musculoskeletal condition in general population, as previous studies revealed an 85% to 90% lifetime incidence of low back pain [1, 2]. Similar to the vast majority of general population, a large number of athletes also experience LBP. Furthermore, athletes of particular types of sports such as ski or gymnastics are at greater risk of low back pain than nonathletes population [3, 4]. The incidence rates of low back pain in athletes have been reported 1% to 30% depending on the specific sport they are involved in [5].

Findings showed that low back pain experienced by athletes would involve and reduce athletic performance [6]. Additionally many athletes who experience low back pain might run into marked disability when they retire from the sports. Thus identification of modifiable risk factors is necessary to reduce the incidence of LBP in athletes. Previous data indicated the athletes' specific LBP patterns and risk factors different from nonathlete population [7]. For example, although research showed that the strength of back muscles is not significantly different between athletes and nonathletes [8, 9], the forces on athlete's back are often greater than nonathletes. Moreover, the athletes sustain these forces for a long time. Furthermore a few sport specific positions or movements can predispose various back-related problems [3, 10]. For instance, athletes involved in sports require repetitive rotation of the back such as ski and gymnastic show a high incidence of spondylolisthesis [11, 12]. Previous data showed that risk factors of low back pain in athletes are multifactorial including sport type [13, 14], repetitive loads [15, 16], and training frequency [7, 17]. However many of suggested risk factors are based on expert opinions, case studies, and unpublished clinical data [4, 18–20] and there is no strong evidence that these factors linked to LBP in athletes. On the other hand some of the published data for such risk factors is controversial. For example, Evans et al. showed that body mass index (BMI) could be a risk factor of athletes' LBP but Kujala et al. could not support their finding [21, 22].

Thus in this study, we conducted a comprehensive review based on prospective studies that evaluated risk factors of LBP in athletes. We believed that pooling these studies revealed evidence for risk factors that would indicate athletes who are at greater risk of future LBP.

## 2. Methods

### 2.1. Search Strategy

To identify studies relating to the risk factors of low back pain among athletes, we searched electronic databases including PubMed, Ovid, Google Scholar, Scopus, and CINAHL for all years available up to June 1, 2015. The search strategy included three elements of outcome problem (low back pain), population (athletes), and possible determinants (risk factors). Keywords used in the search procedure consisted of risk factor^*∗*^, predictor, antecedent, determinant, contributing factor^*∗*^, low back pain, back pain, backache, LBP, athlete^*∗*^, and sport^*∗*^. Key terms were matched to the Medical Subject Headings (MeSH). We also searched the references of all selected articles and key journals related to the topic to help identify studies that could be missed by electronic database searching. Grey literature was also searched to retrieve all the explanatory or evaluation data on the risk factors of LBP in athletes. We did not impose any restriction on language.

### 2.2. Study Selection

All identified citations retrieved by the search strategy were screened to select relevant studies. In the first stage two reviewers independently checked the titles and abstract of all the selected articles. After that, the selection criteria were applied on the full text of all potentially relevant articles. Articles were eligible if they met these criteria:

1. The study which mainly aimed to examine risk factors associated with low back pain in athletes.
2. The study which indicated a longitudinal prospective design (we excluded narrative review, cross-sectional, case control, and single case studies).
3. The study population which included athletes.
4. The study which included athletes diagnosed with symptoms or singes of nonspecific low back pain (we excluded studies on specific low back pain).

In case there was discrepancy between two reviewers, we arranged a consensus meeting or used a third reviewer to make a final decision.

### 2.3. Data Extraction

For each study, data was extracted by the first author and in the regular meetings, all questions resolved by all authors. We extracted information on the study characteristics (design of study, author, and year), population (age, gender, and number), follow-up, sport type, risk factors, and risk estimates (e.g., relative risk) of low back pain. For the purpose of this review, meta-analysis was not applicable since there was a considerable variation among parameters and statistical methods used in studies. Thus we conducted a narrative analysis of the results.

### 2.4. Quality Assessment

The studies that met the inclusion criteria were scored by two independent reviewers (VM and AM). The criteria for quality assessment were based on the Strengthening the Reporting of Observational Studies in Epidemiology (STROBE) (available at the http://www.strobstatement.org/index.php?id=available-checklists) which has been illustrated to be a valid tool for observational studies [23, 24]. The modified checklist included nine items from original STROBE list (Table 2). These items were scored as “1” (positive) and “0” (negative). As shown in Table 2, a total quality score was calculated for each study. If the quality score was 70% of the maximum score or more, we defined the study as a high quality, and a study was considered as low quality if the score was less than 70%. A consensus between two reviewers was met and third author were used to sort out differences.

### 2.5. Level of Evidence

To determine the strength and quality of reported risk factors, level of evidence was set based on the number, quality, and outcome of the studies as follows [25, 26]. If there are generally consistent findings in multiple (≥2) high quality studies, evidence will be strong. If there are generally consistent findings in one high quality study and one or more low quality studies or in multiple (≥2) low quality studies, evidence will be moderate. If only one study exists or findings in multiple (≥2) studies are inconsistent, evidence will be insufficient.

## 3. Results

**Figure 1:**
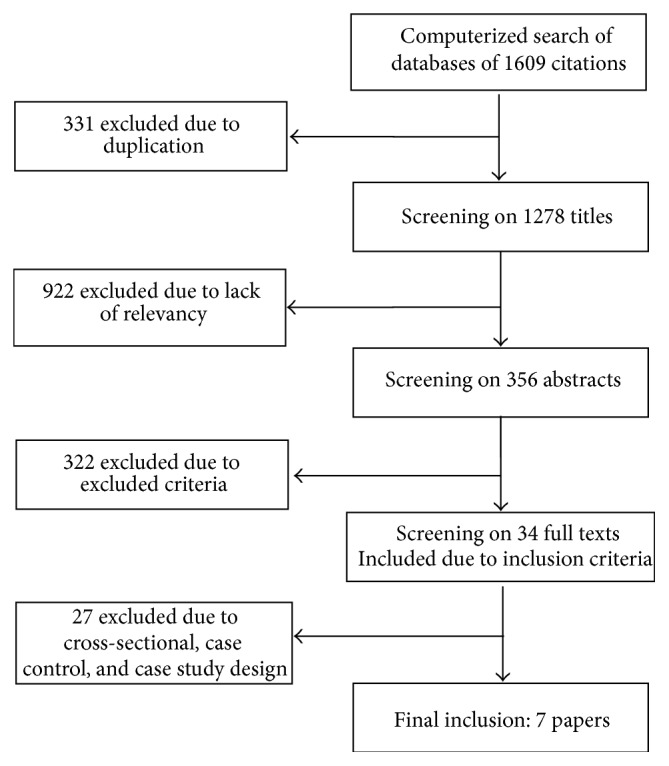
Flowchart of studies selection.

The search in all databases yielded 1608 papers (Figure 1). After removing duplicates, the main reason for exclusion was that the articles were not sport specific. Thus, 356 articles were extracted for abstract review; in time, 34 papers were recognized as relevant for the full-text review. Of thirty four papers, 27 articles were excluded because they indicated case control, cross-sectional, or case study design and consequently we identified seven eligible studies that reported risk factors of LBP. Figure 1 displays the process of selecting the studies.

### 3.1. Study Characteristics

Table 1 shows selected characteristics of included studies. Three of these studies were published before the year 2000 and fourth of them were published after 2000. The studies' sample size was between 14 and 257. In two studies, participants were just male, but the other studies examined both male and female athletes. Low back pain was measured by a questionnaire or clinical observation. The period of follow-up ranged from one to three years.

### 3.2. Quality Assessment

Two reviewers scored the methodological quality of the included articles studies (Table 3). Disagreement between reviewers was discussed and resolved by consensus. The quality score of the studies ranged from 44% to 77%. Four articles had a quality score ≥70%, thus considered as being of high quality, and three had a score <70%, thus considered as being of low quality.

### 3.3. Risk Factors for Low Back Pain

#### 3.3.1. Flexibility

Six variables for flexibility in four studies were measured (Table 4). Hip flexor tightness, decreased lumbar flexion, and extension and forward bending indicated significant differences between athletes with and without LBP. One high quality and one low quality studies reported that flexor tightness was significantly and negatively associated with LBP [21, 22]. Also, flexor tightness was not associated with low back pain in one low quality study [27]. Decreased lumbar flexion and extension were significant risk factors for LBP in two high quality studies [21, 28] but were not associated with low back pain in one low quality study [22]. These studies assessed lumbar sagittal flexibility using a modified version of the flexicurve technique introduced by Tillotson and Burton [29]. The flexicurve (a malleable band of metal) was placed on the lumbar spine in maximal flexion, extension, and habitual posture standing, and then based on the obtained tangent angles in these situations, flexion and extension would be calculated. Furthermore modified-modified Schober method has been used to evaluated lumbar flexion and extension (for more details, see [30]). One high quality study identified that forward bending was associated with LBP in male athletes. Other variables (such as trunk side bending and hamstring tightness) were not significantly associated with LBP in athletes. Hjelm et al. in a low quality study examining range of motion (ROM) showed that difference range of motion >10° in dominant and nondominant side as a risk factor for LBP. This evaluation was performed in neck, shoulder, and elbow joints. Finally, decreased lateral flexion of the neck at the dominant side was considered as significant risk factor [31].

#### 3.3.2. Strength

For muscle strength, two studies evaluated trunk extensor strength in athletes: one was low quality [22] and another was high quality [32]. However their findings showed that this variable was not significantly different between athletes with and without LBP. In addition, hip extensor strength in one high quality study was measured [32]. The results of this study displayed that there is no significant difference for hip extensor strength between athletes with and without LBP.

#### 3.3.3. Endurance Test

Two studies reported endurance test scores related to low back pain in athletes. These variables were trunk extensor endurance, trunk flexor endurance, and side bridge endurance. Side bridge endurance strength was significantly associated with low back pain from one low quality study [22]. There were no significant associations between LBP and trunk flexor and extensor endurance based on one low quality study [21, 22].

#### 3.3.4. Previous Low Back Pain

Previous LBP as a risk factor was examined in two high quality studies [21, 33] and one low quality study [27]. One of these studies showed that previous low back pain at the baseline was predictor of LBP during follow-up among female athletes [21]. Burdorf et al. described that the various subcategories of previous LBP were strong predictors for LBP [33]. However Nadler et al. in their study found no different significance between previous low back pain and LBP [27].

#### 3.3.5. Previous Injury

Previous back injury was examined and reported as a main risk factor of LBP in athletes by one low quality study. Indeed patellofemoral syndrome and ankle sprain were significantly related to low back pain [27].

#### 3.3.6. Anthropometric Characteristics

Anthropometric data were reported in all studies, but only four evaluated the association between anthropometric characteristics and risk of low back pain in athletes. Two high quality studies showed that the athletes with higher body weight were at higher risk for LBP [21, 28]. From other anthropometric factors, BMI was a significant risk factor for low back pain in two studies. One was high quality [28] and another was low quality [22]. No significant associations were found for age, sex, height, upper body mass, and lower body mass with LBP [21, 28, 33].

#### 3.3.7. Sport Related Factors

Participation in other sports was assessed in two studies [31, 33], but their results indicated no association with LBP. One of these studies was low quality [31]. Amount of playing per week was significantly associated with low back pain in one low quality study [31]. One low quality study evaluated active year in sport as a potential risk factor, but its findings showed no significant association with LBP [31].

## 4. Discussion

This is the first known systematic review of existing data on risk factors for low back pain in athletes. Results showed that there were general and sport specific risk factors that can predict those athletes who are at greater risk of LBP. We presented findings in two paragraphs including sport specific and general risk factors in following lines.

To address sport specific risk factors, our finding illustrated that decreased lumbar ROM either flexion or extension is a strong risk factor for LBP in athletes [21, 22, 28]. However in contrast, a review study on working or general population indicated that the lumbar flexion is not an independent risk factor of low back pain [34]. This contrast may be related to differences between characteristics of athlete and nonathlete participants. We found moderate evidence for flexor tightness as a risk factor of low back pain in athletes [21, 22, 27], but there was no sufficient evidence that hamstring tightness or forward bending contributed to increasing the risk of low back pain in athletes [21, 22, 28]. Previous data from general populations also replicated these findings [21, 34]. Examining other potential risk factors showed that there was moderate evidence to indicate a lack of association between trunk or hip extensor strength and low back pain in athletes [22, 32]. This finding is in line with a study on working population that strongly showed that there is no association between muscle strength and LBP [34]. In agreement with Hamberg-van Reenen et al., our finding confirmed that there was no sufficient evidence for associations between LBP and trunk flexor or extensor endurance [21, 22, 34]. Although there was no adequate evidence to indicate the role of injury in LBP, our findings showed strong evidence for association between previous LBP and future low back pain in athletes [21, 27, 33]. We also found moderate evidence for no association between LBP and participation in other sports. Eventually about the effects of active years in sport and training frequency, there were still insufficient evidences.

Furthermore general or background characteristics including age, sex, weight, height, and BMI were examined by studies as potential variables which might increase risk of LBP in athletes. We found strong evidence for weight (higher body weight was a risk for LBP) and moderate evidence for BMI (higher BMI was a risk for LBP) as risk factors of LBP in athletes. There was also strong evidence to indicate that height is not a risk factor for LBP but insufficient evidence to indicate age and sex as risk factors for LBP in athletes. Based on data from general population there was no association between body weight and low back pain [35]; however Balagué et al. showed that increase in age, increased height, and female gender were significantly associated with low back pain [35].

Overall, the findings of previous reviews on general population were in part different from our findings. A good explanation may be due to differences between athletes and nonathletes' characteristics [36]. Sport type, level of competition, and training frequency can influence the association of LBP with potential risk factors among athlete population [37, 38]. Furthermore, we only included longitudinal studies that might further explain observed differences between these reviews.

### 4.1. Quality Assessment

Of seven studies that were considered in this review, three had low and four had high methodological quality. We modified the STROBE that is a valid quality assessment for observational studies based on the assumption that the quality items such as method of assessment, adjustment for confounders, and estimates of relative risk have more effect on the level of evidence than the other items. All items were equally weighted for this systematic review. Method of measurement and reporting the number of outcome events were the strength point of these studies. However few studies reported relative risk estimates for LBP or how confounders were included in their analysis. These were the main limitation of the studies reviewed.

### 4.2. Limitations

There were a few limitations to these studies. First, participants were included from different sports; thus the results were not clearly specified for each sport [21, 28]. Second, the most common shortcoming of studies was the lack of adequate data presentation. Despite the fact that the studies were longitudinal, most of them did not report the relative risk estimates to explain the association of risk factor with LBP; they used a variety of statistical methods instead. As a result, meta-analysis was not applicable. Also, including only longitudinal studies and excluding the cross-sectional or case control studies may reduce our pool of risk factors. In this review, we just considered nonspecific low back pain; thus the result of this review cannot address the other kind of low back pain in athletes. Also, since existing data were available from limited numbers of sports, many risk factors related to other sports have not been yet assessed. We used an adapted quality assessment tool; and one can argue that the selected items can bias the quality scores. Accordingly we have provided a few recommendations for future research. First, future studies are supposed to focus on risk factors in sports where LBP is extremely common such as volleyball. We strongly recommend examining psychological, behavioural, and social variables that may increase the risk of LBP in athletes. It is worth noting that psychosocial factors are important in development of chronic problems. A poor social environment in addition to inadequate personal resources to handle the demands of sports environment may increase stress response and in turn increase the muscle tone or musculoskeletal symptoms in athletes. This may lead to enhancement of the perception or reporting symptoms or a reduction of the ability to deal with LBP related symptoms. Indeed competition may lead to significant psychological stress, which can serve to reinforce the effect of psychological stressors. Finally we recommend not only conducting longitudinal studies but also using a rigorous multivariate analysis adjusting for important confounders to provide a more quantifiable basis for associations.

## 5. Conclusion

Although there were many potential risk factors for low back pain in athletes, we conclude strong evidence for only a few risk factors of low back pain including decreased lumbar flexion or extension, previous low back pain, and high body weight. This review revealed a high heterogeneity of participants, measured exposures, and statistical techniques used in studies of LBP risk factors in athletes. To develop preventive interventions that decrease risk of LBP in athletes, we need more high-quality evidence on the possible risk factors of LBP.

## Figures and Tables

**Table 1:** Study characteristics of included studies.

	Author	Sport	Follow-up	Sample size	Age	Groups	Examined risk factor	Conclusion
1	Burdorf et al., 1996 [33]	Golf	1 year	Male: 196	20–60	LBP (134) Non-HLB (72)	Age (years) Previous low back pain more than once and lasting >3 months Involvement in other sports Absence from work because of previous back pain	Previous back pain was strong predictor

2	Evans et al., 2005 [22]	Golf	3 years	Male: 14	18–35 yr	With LBP (8) Without LBP (6)	Anthropometric variables, flexibility, muscle strength, and muscle endurance	BMI, hip flexor length, and asymmetry on the side bridge endurance test were significantly correlated No other anthropometric or flexibility test was significantly correlated with reports of LBP

3	Hjelm et al., 2012 [31]	Tennis	2 years	Total: 55 Male: 35 Female: 25	12–18	Injured: 39 Uninjured: 16	Single and double play: (>6 versus <6 h/week) Previous injury of the back lateral flexion of the neck at the dominant and nondominant side (decreased) Rack side and length (normal/extra length) Tennis hours/year Participating in other sports >10° difference between dominant and nondominant ROM of (neck, shoulder, and elbow)	Previous injury to the back and playing tennis more than 6 h per week and decreased lateral flexion of the neck at the dominant side were reported as risk factors for back pain

4	Kujala et al., 1994 [21]	Soccer Ice hockey Gymnastics Skating Ballet	1 year	Total: 119 Male: 52 Female: 67	10.3–13.3	With LBP: 24 Without LBP: 95	Lumbar flexion and extension, forward and side bending, hip flexor tightness, hamstring tightness, back and abdominal endurance	Decreased lumbar flexion and hip flexor tightness (in male) and high body weight and previous LBP in female were predictor

5	Kujala et al., 1997 [28]	Soccer Ice hockey Gymnastics Skating	3 years	Baseline: 116 Follow-up: 98 Male: 50 Female: 48	10.3–13.3	With LBP: 35 Without LBP: 63	Anthropometric variables and lumbar mobility	Predictor: low maximum extension of the lumbar

6	Nadler et al., 1998 [27]	Soccer Football Baseball Track Tennis Swimming Basketball Volleyball Softball	1 year	Total: 257 Male: 170 Female: 87		With LBP: 31 Without LBP: 109	Lower extremity injuries flexor tightness	Significant difference: in lower extremity injuries and previous LBP

7	Renkawitz et al., 2006 [32]	Tennis		Total: 82 Male: 51 Female: 31	33	With LBP: 46 Without LBP: 36	Spinal mobility and muscular flexibility of the lower back	Neuromuscular imbalance: direct relationship between LBP
							Isometric voluntary maximum trunk extension strength	Maximum isometric trunk extension strength was not predictor

**Table 2:** Standard checklist for assessment of methodological quality of prospective cohort studies.

Item	Score: no = 0/yes = 1
(1)	Describe the setting, locations, and relevant dates, including periods of recruitment, exposure, follow-up, and data collection

(2)	Give the eligibility criteria and the sources and methods of selection of participants. Describe methods of follow-up

(3)	Clearly define all variables (outcomes and exposures) considered for and included in the analysis. Give diagnostic criteria, if applicable

(4)	For each variable of interest, give sources of data and details of methods of assessment (measurement)

(5)	Describe all statistical methods, used to examine subgroups, interactions, and control for confounding

(6)	If applicable, explain how loss to follow-up was addressed

(7)	Report descriptive data; give characteristics of study participants (e.g., demographic, clinical, and social) and information on exposures and potential confounders

(8)	Report numbers of outcome events or summary measures over time

(9)	Give unadjusted estimates and, if applicable, confounder-adjusted estimates and their precision (e.g., 95% confidence interval). Make clear which confounders were adjusted for and why they were included

**Table 3:** The results of quality assessment of included studies.

Study	Items of quality assessment^*∗*^									Total (%)
	1	2	3	4	5	6	7	8	9	
Kujala et al., 1994 [21]	1	0	1	1	1	1	1	1	0	77
Kujala et al., 1997 [28]	1	1	0	1	1	0	1	1	1	77
Burdorf et al., 1996 [33]	0	1	1	0	1	1	1	1	1	77
Nadler et al., 1998 [27]	0	1	1	1	0	0	0	1	0	44
Evans et al., 2005 [22]	1	1	1	1	0	0	1	1	0	66
Renkawitz et al., 2006 [32]	1	1	1	1	1	0	1	0	1	77
Hjelm et al., 2012 [31]	0	0	0	1	1	1	1	1	0	55

^*∗*^The items are observable on Table 1.

**Table 4:** The results of prospective studies on risk factors of low back pain in athletes.

Risk factor	Number	Study	Association	Sample showing association		Level of evidence
				Yes	No	
Age	1	[33]	0		1/1	Insufficient

Body weight	2	[21, 28]	+	2/2		Strong

BMI	2	[22, 28]	−	2/2		Moderate

Height	2	[21, 28]	0			Strong

Sex	1	[21]	0		1/1	Insufficient

Involvement in other sports	2	[31, 33]	0		2/2	Moderate

Active year	1	[31]	0		1/1	Insufficient

Previous LBP	3	[21, 33]	−	2/3	1/3	Strong
		[27]	0			

Previous back injury	1	[31]	−	1/1		Insufficient

Previous lower extremity injury	1	[27]	−	1/1		Insufficient

Lumbar flexion (decreased ROM)	3	[21, 28]	−	2/3	1/3	Strong
		[22]	0			

Lumbar extension (decreased ROM)	3	[21, 28]	−	2/3	1/3	Strong
		[22]	0			

hip flexor length (tightness)	3	[21, 22]	−	2/3	1/3	Moderate
		[27]	0			

Forward bending	1	[21]	+	1/1		Insufficient

Side bridge endurance test	1	[22]	−	1/1		Insufficient

Lateral flexion of the neck at the dominant side (decreased)	1	[31]	−	1/1		Insufficient

hamstring length (tightness)	3	[21, 22, 28]	0		3/3	Strong

Trunk flexor endurance	2	[21, 22]	0		2/2	Moderate

Trunk extensor endurance	2	[21, 22]	0		2/2	Moderate

trunk extensor strength	2	[22, 32]	0		2/2	Moderate

Hip extensor strength	1	[32]	0		1/1	Insufficient

Lateral flexion of the neck at the nondominant side (decreased)	1	[31]	0		1/1	Insufficient

Rack side et length (normal/extra length)	1	[31]	0		1/1	Insufficient

>10° difference between dominant and nondominant total shoulder rotation	1	[31]	0		1/1	Insufficient

>10° difference between dominant and nondominant side in flexion of the shoulder joint	1	[31]	0		1/1	Insufficient

>10° difference between dominant and nondominant side in extension of the elbow joint	1	[31]	0		1/1	Insufficient

>10% difference between forehand and backhand medicine ball tosses	1	[31]	0		1/1	Insufficient

+: positive association, −: negative association, and 0: without association.
